# Experimental Alcohol-Related Peripheral Neuropathy: Role of Insulin/IGF Resistance

**DOI:** 10.3390/nu4081042

**Published:** 2012-08-17

**Authors:** Van Anh Nguyen, Tran Le, Ming Tong, Michelle Mellion, James Gilchrist, Suzanne M. de la Monte

**Affiliations:** 1 Department of Medicine, Rhode Island Hospital, Alpert Medical School of Brown University, 55 Claverick Street, Providence RI 02903, USA; Email: va.nguyen87@gmail.com (V.A.N.); tran.le09@gmail.com (T.L.); ming_tong_ming@yahoo.com (M.T.); 2 Department of Neurology, Rhode Island Hospital, Alpert Medical School of Brown University, 593 Eddy Street, Providence RI 02903, USA; Email: mmellion1@lifespan.org (M.M.); JGilchrist@lifespan.org (J.G.); 3 Departments of Neuropathology/Pathology, Neurology, Neurosurgery, and Medicine, Rhode Island Hospital, Alpert Medical School of Brown University, 55 Claverick Street, Providence RI 02903, USA

**Keywords:** alcoholic peripheral neuropathy, insulin resistance, nerve conduction, experimental animal model, gene expression, demyelination, nutritional deficiency

## Abstract

The mechanisms of alcohol-related peripheral neuropathy (ALPN) are poorly understood. We hypothesize that, like alcohol-related liver and brain degeneration, ALPN may be mediated by combined effects of insulin/IGF resistance and oxidative stress. Adult male Long Evans rats were chronically pair-fed with diets containing 0% or 37% ethanol (caloric), and subjected to nerve conduction studies. Chronic ethanol feeding slowed nerve conduction in the tibial (*p* = 0.0021) motor nerve, and not plantar sensory nerve, but it did not affect amplitude. Histological studies of the sciatic nerve revealed reduced nerve fiber diameters with increased regenerative sprouts, and denervation myopathy in ethanol-fed rats. qRT-PCR analysis demonstrated reduced mRNA levels of insulin, IGF-1, and IGF-2 polypeptides, IGF-1 receptor, and IRS2, and ELISAs revealed reduced immunoreactivity for insulin and IGF-1 receptors, IRS-1, IRS-4, myelin-associated glycoprotein, and tau in sciatic nerves of ethanol-fed rats (all *p* < 0.05 or better). The findings suggest that ALPN is characterized by (1) slowed conduction velocity with demyelination, and a small component of axonal degeneration; (2) impaired trophic factor signaling due to insulin and IGF resistance; and (3) degeneration of myelin and axonal cytoskeletal proteins. Therefore, ALPN is likely mediated by molecular and signal transduction abnormalities similar to those identified in alcoholic liver and brain degeneration.

## 1. Introduction

Alcohol-related polyneuropathy (ALPN) is a chronic and potentially debilitating disease that can be associated with sensory, motor, and autonomic nerve dysfunctions. In addition to discomfort and functional impairments, secondary effects of ALPN include, repeated injury to the extremities, infection, head trauma from falls, bowel, bladder, and sexual dysfunction, and in some cases, permanent disability [1]. Clinically significant ALPN occurs more frequently than appreciated, with rates as high as 66% among severe alcoholics [2,3,4,5,6,7]. Regarding the pathogenesis of ALPN, considerable attention has been paid to the contributions of malnutrition, particularly thiamine deficiency, because thiamine deficiency often complicates alcohol-related diseases, and thiamine deficiency alone can cause peripheral neuropathy [8,9,10,11]. Alcoholics are prone to thiamine deficiency because: (1) the nutritional support is often marginal or wholly inadequate; (2) alcohol impairs thiamine absorption through the gastrointestinal tract [12,13,14] and its utilization in tissues [15,16]; and (3) alcohol inhibits hepatic storage [15,16] and phosphorylation of thiamine, reducing the availability of thiamine pyrophosphate, the active form [17,18,19]. However, the concept that ALPN is mainly caused by thiamine deficiency has lost steam because in controlled clinical trials, ALPN was not significantly abated or reversed by thiamine repletion [20,21,22]. Therefore, attention should be focused on how alcohol toxicity either alone or in combination with thiamine deficiency, promotes ALPN. 

Although ALPN and thiamine deficiency neuropathies both cause symmetric sensorimotor deficits involving lower extremities with evidence of axonal degeneration [8,11,23], several features of ALPN are distinctive [8,9,24,25]. ALPN is associated with slowly progressive, sensory-dominant deficits with burning pain and superficial loss of sensation, and damage to mainly small fibers, including irregular segmental demyelination and remyelination [9,24,25], whereas thiamine deficiency mainly causes motor-dominant neuropathies that result in acutely progressive deficits in superficial and deep sensation, due to degeneration of large fiber axons and subperineurial edema [8,9,10,24]. On the other hand, studies have shown that nutritional/thiamine deficiencies can contribute to the clinical, electrophysiological, and neuropathological manifestations of ALPN [8,9,23,24,25], thereby accounting for the partial responses to vitamin B therapy [20,22]. Altogether, these findings suggest that additional information is needed to better understand the pathogenesis of ALPN in order to develop new strategies for treatment and possibly prevention of disease. Clues may be provided from studies of other alcohol-related diseases.

Emerging data suggest roles for impaired insulin and insulin-like growth factor (IGF) signaling mechanisms and increased oxidative stress in the pathogenesis of alcohol-related diseases of liver and brain in both humans and experimental animals [26,27,28,29]. Insulin/IGF resistance and oxidative stress promote cell loss and neurodegeneration [30]. In addition, insulin/IGF have important roles in regulating myelin maintenance in both peripheral and central nervous systems [31,32,33,34,35]. In addition, an established feature of alcohol-related brain disease, both in humans and experimental animal models is white matter atrophy and reduced myelin gene expression [36,37,38]. In the brain, oligodendrocytes maintain myelin via insulin/IGF signaling [32,39,40,41,42]. Similarly, in the peripheral nervous system, Schwann cells utilize IGF signaling for myelinogenesis and myelin maintenance [33,43]. Little is known about the role of Schwann cell insulin/IGF resistance as a mediator of ALPN. Since pharmaceutical agents such as peroxisome-proliferator activated receptor (PPAR) agonists that can restore insulin/IGF sensitivity while reducing oxidative stress, already exist and have proven benefits for treating alcohol-related liver and brain disease [44,45,46], determining whether ALPN is also mediated by impaired insulin/IGF signaling would provide opportunities to explore novel and alternative treatments for this disease. Our working hypothesis is that chronic alcohol abuse causes ALPN by impairing insulin/IGF signaling in peripheral nerve elements. 

## 2. Experimental Section

### 2.1. Materials

The bicinchoninic acid (BCA) kit to measure protein concentration was purchased from Pierce Chemical Co. (Rockford, IL). Histochoice fixative was purchased from Amresco, Inc. (Solon, OH). Maxisorp 96-well plates used for enzyme-linked immunosorbent assays (ELISAs) were from Nunc (Thermo Fisher Scientific; Rochester, NY). Superblock-TBS, and horseradish peroxidase conjugated antibodies were from Pierce Chemical Co. (Rockford, IL). All other monoclonal and polyclonal antibodies and immunodetection reagents for ELISAs were purchased from Abcam (Cambridge, MA), Upstate (Billerica, MA), Vector Laboratories (Burlingame, CA), Invitrogen (Carlsbad, CA) or Chemicon (Temecula, CA). Fine chemicals were purchased from CalBiochem (Carlsbad, CA), or Sigma-Aldrich (St Louis, Mo.). QIAzol Lysis Reagent for RNA extraction and QuantiTect SYBR Green PCR Mix were obtained from Qiagen, Inc. (Valencia, CA). The AMV 1st Strand cDNA Synthesis Kit was purchased from Roche Applied Science (Indianapolis, IN). Synthetic oligonucleotides used in quantitative polymerase chain reaction (qPCR) assays were purchased from Sigma-Aldrich Co. (St. Louis, MO). The Stereologer system used for image analysis was purchased from the Stereology Resource Center (Chester, MD). Neuroline subdermal needle electrodes used in nerve conduction studies were purchased from Ambu (Glen Burnie, MD). 

### 2.2. Chronic Ethanol Exposure Model

Adult male (~200–250 g) Long Evans rats (Harlan Sprague Dawley, Inc., Indianapolis, Indiana) were pair-fed with isocaloric liquid diets (BioServ, Frenchtown, NJ) containing 0% (*N* = 8) or 37% (*N* = 13) ethanol for 8 weeks [26,29,47]. Two weeks prior to initiating the experiment, rats were adapted to the liquid diets by incrementing the ethanol content from 0% to 11.8%, 23.6% and then 37% of the caloric content. Controls were adapted to ethanol-free liquid diets over the same period. Rats were monitored daily to ensure adequate nutritional intake and maintenance of body weight. Blood alcohol levels were measured at 8 AM using the Analox GM7 apparatus (Analox Instruments USA, Lunenburg, MA) according to the manufacturer’s protocol. At the end of the experiment, the rats were sacrificed by isofluorane inhalation, and peripheral nerve and skeletal muscle (gastrocnemius) tissues were snap-frozen in a dry ice/methanol bath for later protein and RNA studies, or immersion fixed in Histochoice for histological studies. Throughout the experiment, rats were housed under humane conditions and kept on a 12-h light/dark cycle with free access to food. All experiments were performed in accordance with protocols approved by Institutional Animal Care and Use Committee at the Lifespan-Rhode Island Hospital, and they conform to guidelines established by the National Institutes of Health. 

### 2.3. Electrophysiology

Nerve conduction studies (NCS) were performed during the 7th week of liquid diet feeding. Under sodium pentobarbital anesthesia (40–50 mg/kg), nerve conduction velocity and amplitude were measured in the plantar, tibial, and peroneal nerves with a Nicolet Biomedical Inc. Viking IV Electromyography System using standard filter settings for motor, mixed, and sensory NCS. Sensory nerve action potentials were recorded [48], and negative peak latency and peak-to-peak amplitude were measured. Compound motor action potentials were measured after delivery of a supra-maximal stimulus to obtain the maximum response. Latencies and peak-to-peak amplitudes for all stimulations were measured, and velocities were calculated by dividing distance by latency [49]. At the end of the study, segments of peripheral nerve and skeletal muscle (contra-lateral to the NCS site to avoid artifacts) were snap-frozen for protein and RNA studies, or immersion fixed for histology and morphometric analysis.

### 2.4. Histology and Morphometric Analysis

For histopathological studies, sections of peripheral nerve were fixed in Histochoice, embedded in paraffin, and sections (5 µm thick) were stained with Luxol Fast Blue/Hematoxylin and Eosin. In addition, Histochoice fixed segments of peripheral nerve were rinsed in 0.15 M sodium cacodylate buffer and placed in 2.5% glutaraldehyde in 0.15 M sodium cacodylate buffer for 1 h. After 3 rinses in cacodylate buffer, the tissues were post-fixed in buffered 1% osmium tetroxide for 1 h at 4 °C. Tissues were rinsed in cacodylate buffer, dehydrated through a graded acetone series, and infiltrated with Spurr’s epoxy resin. After overnight polymerization at 70 °C, 1 µm thick sections were cut with a Reichert Ultracut S ultra microtome. Sections were stained with methylene blue-azure II and examined and photographed by light microscopy. Morphometric analysis to measure nerve fiber diameters was performed at 600× oil magnification using the disector, point counting, and nucleator probes of the Stereologer program to determine density and diameter of fibers. In addition, Hematoxylin and Eosin stained sections of skeletal muscle were used to measure myofiber diameter with the Sterologer program. All analyses were performed under code.

### 2.5. Quantitative Reverse Transcriptase Polymerase Chain Reaction (qRT-PCR) Assays of Gene Expression

Total RNA was isolated from peripheral nerve using the EZ1 RNA Universal Tissue Kit and the BIO Robot EZ1 (Qiagen Inc., Valencia, CA). RNA was reverse transcribed with random oligonucleotide primers and the AMV First Strand cDNA synthesis kit, and the resulting cDNAs were used to measure gene expression by qPCR with gene-specific primer pairs as reported elsewhere [26,29,47]. The Master ep realplex instrument and software (Eppendorf AG, Hamburg, Germany) were used to detect amplified signals in triplicate reactions. Relative mRNA abundance was calculated from the ng ratios of mRNA to 18S rRNA measured in the same samples, and those data were used for inter-group comparisons. 

### 2.6. Enzyme-Linked Immunosorbant Assay (ELISA)

Tissues were homogenized in radioimmunoprecipitation assay buffer containing protease and phosphatase inhibitors [26,29,47]. Direct binding ELISAs were performed in 96-well plates [26,29]. In brief, proteins (40 ng/100 µL) adsorbed to well bottoms by over-night incubation at 4 °C were blocked with 3% BSA in Tris buffered saline (TBS), and then incubated with primary antibody (0.2–1.0 µg/mL) for 1 h at room temperature. Immunoreactivity was detected with horseradish peroxidase (HRP)-conjugated secondary antibody and Amplex Red soluble fluorophore. Fluorescence was measured (Ex 530/Em 590) in a SpectraMax M5 microplate reader. Binding specificity was monitored in parallel control incubations that included non-relevant antibodies, or had the primary or secondary antibody omitted. Immunoreactivity was normalized to protein content in parallel wells as determined with the NanoOrange Protein Quantification Kit.

### 2.7. Statistical Analysis

Data depicted in box plots reflect group median, 95% confidence interval limits (upper and lower boundaries of boxes) and range (whiskers), while tabulated data reflect means ± SEMs for each group. Intergroup comparisons were made using Student *T*-tests. Data were analyzed using GraphPad Prism 5 software (GraphPad Software, Inc., San Diego, CA). Significant *p*-values (<0.05) are shown within the graph panels or included in the tables.

## 3. Results

### 3.1. General Effects of Ethanol Feeding

The control and ethanol-fed rats gained weight continuously throughout the study, and although the ethanol-fed rats weighed less than control, the differences in mean weight were not statistically significant (Table 1). The liquid diets were nutritionally complete, including ample multi-B vitamin supplementation. Fresh food was provided daily to ensure high quality and consistent feedings. As expected, the mean blood alcohol concentration was elevated in ethanol-fed rats and non-detectable in controls. Throughout the study, the rats in both groups remained in excellent health, self-groomed, and were physically active, manifesting no overt signs of motor weakness or discomfort. 

**Table 1:** Body weight gain and blood alcohol concentrations. The mean ± S.D. of initial, final, and net percentage increase in body weight are listed (*N* = 8 Control; *N* = 13 Ethanol). Blood alcohol concentrations are indicated. Inter-group comparisons were made by Student *T*-tests.

Variable	Control	Ethanol	*p*-Value
Initial body wt (g)	361.0 ± 31.5	353.1 ± 24.7	
Final body wt (g)	454.1 ± 18.4	423.8 ± 39.6	
% Body wt gain	21.04 ± 6.2	19.59 ± 8.4	
Blood alcohol (mg/dL)	2.62 ± 0.9	129.9 ± 12.0	*p* < 0.0001

### 3.2. Effects of Ethanol on Nerve Conduction

Nerve conduction studies were performed on the plantar (sensory), tibialis (motor), and peroneal (motor) nerves. Recordings from motor nerves were made at the knee and ankle. Those studies demonstrated similar mean amplitudes of nerve conduction in sensory and motor nerves of control and ethanol-fed rats, and similar mean latencies and conduction velocities in plantar nerves of control and ethanol-fed rats (Table 2). In contrast, mean conduction velocities were significantly reduced in the tibialis nerve of ethanol-fed relative to control rats (*p* = 0.0021). 

**Table 2:** Nerve Conduction Studies. Summary data reflect the mean ± S.E.M of latencies and amplitudes for control (*N* = 8) and ethanol-fed (*N* = 13) rats. Inter-group comparisons were made using Student *T*-tests. The longer tibialis nerve latency in ethanol-fed rats reflects slowed responses to stimulation, possibly due to demyelination or functionally impaired conductivity. * *p* = 0.0021.

Nerve	Control	Ethanol	Control	Ethanol
	Latency	Latency	Amplitude	Amplitude
Plantar	0.91 ± 0.01	0.96 ± 0.03	77.29 ± 8.92	78.49 ± 11.18
Tibialis (Ankle)	1.24 ± 0.06	1.18 ± 0.08	4.37 ± 1.28	4.85 ± 0.45
Tibialis (Knee)	1.69 ± 0.05	2.00 ± 0.07 *	3.97 ± 1.13	4.58 ± 0.50
Peroneal (Ankle)	1.23 ± 0.07	1.11 ± 0.08	5.68 ± 0.81	5.76 ± 0.86
Peroneal (Knee)	1.71 ± 0.09	1.81 ± 0.08	4.94 ± 0.58	5.42 ± 0.83

### 3.3. Histopathology of Alcohol-Related Polyneuropathy

**Figure 1:**
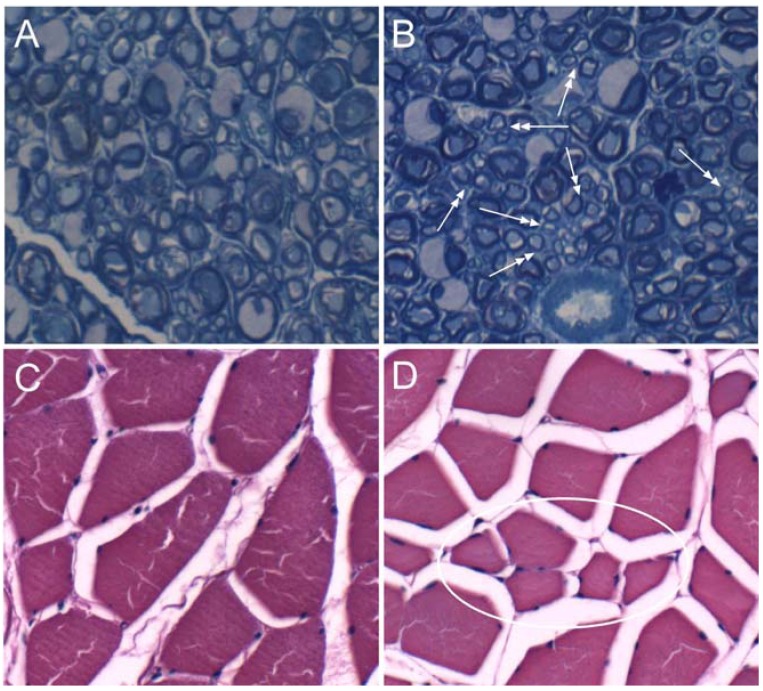
Chronic ethanol feeding causes peripheral neuropathy and denervation myopathy. Adult Long Evans rats were fed with (**A**, **C**) 0% or (**B**, **D**) 37% ethanol-caloric content liquid diets for 8 weeks. (**A**, **B**) Sciatic nerves and (**C**, **D**) gastrocnemius muscles were harvested immediately upon sacrifice. Peripheral nerves were fixed in glutaraldehyde and embedded in Epon. 1 µm thick sections stained with Toluidine blue and photographed at 600× original magnification. Note the relative uniformity of myelinated fibers in the (**A**) control nerve compared with the presence of (**B**) multiple clusters of small thinly myelinated fibers (sprouts) in the nerve from an ethanol-fed rat (arrows). Gastrocnemius muscle biopsies were fixed in Histofix and embedded in paraffin. Hematoxylin and eosin stained, 5 µm-thick sections were photographed at 200× original magnification. Note generally smaller sizes of myofibers in the (**D**) ethanol-fed compared with the (**C**) control rat, and the small cluster of atrophic, somewhat angulated myofibers (encircled) in the ethanol-exposed muscle, corresponding with effects of denervation.

Histological studies of sciatic nerves revealed patchy demyelination in ethanol-fed rats. Correspondingly, the 1 µm thick, Epon-embedded Toluidine blue stained sections revealed numerous clusters of small sprouts and many thinly myelinated axons in ethanol-exposed peripheral nerves (Figure 1). Morphometric analysis of the 1-µm thick sections revealed significantly smaller mean fiber diameters in ethanol-fed relative to control rats (Table 3). Histological sections of gastrocnemius muscles demonstrated mild denervation myopathy characterized by the presence of individual and small groups of angulated atrophy fibers in ethanol-exposed but not control rats (Figure 1). 

Ethanol-associated myofiber atrophy was confirmed by morphometric analysis, which demonstrated significantly smaller mean myofiber diameters in the ethanol-exposed relative to control muscles (Table 3).

**Table 3:** Chronic ethanol feeding causes muscle atrophy. Gastrocnemius muscles were fixed and embedded in paraffin. H&E stained sections were subjected to image analysis to measure myofiber diameters and cross-sectional areas. At least 200 fibers per specimen were measured. Inter-group comparisons were made using Student *T*-tests.

Variable	Control	Ethanol	*p*-Value
Sciatic nerve diameter (µm)	16.48 ± 1.92	11.48 ± 0.91	0.007
Gastrocnemius fiber diameter (µm)	45.83 ± 5.08	25.34 ± 4.38	0.0001
Gastrocnemius fiber area (µm^2^)	2338.64 ± 643.04	695.08 ± 163.35	0.0005

### 3.4. Ethanol Effects on Insulin/IGF Signaling Pathway Genes-qRT-PCR Studies

**Figure 2:**
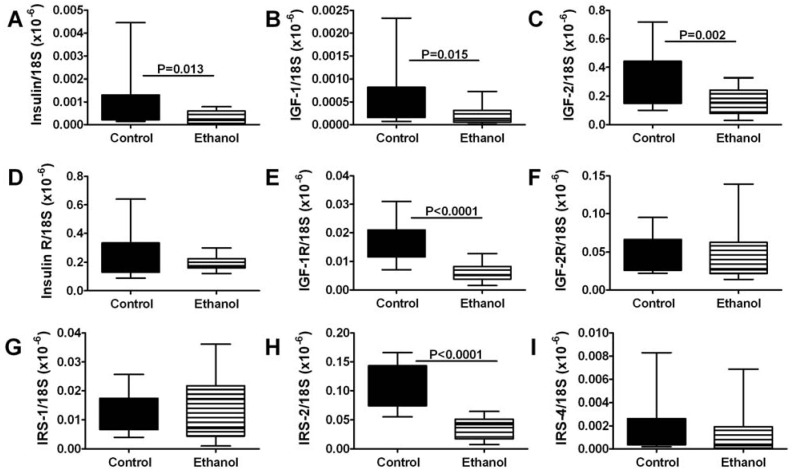
Effects of chronic ethanol feeding on insulin/IGF signaling network gene expression in peripheral nerve. Rats were fed for 8 weeks with liquid diets containing (**A**,**C**) 0% or (**B**,**D**) 37% ethanol (caloric) liquid diets. Sciatic nerves (*N* = 8/group) were used to measure mRNA to (**A**) insulin, (**B**) IGF-1, (**C**) IGF-2, (**D**) insulin receptor, (**E**) IGF-1 receptor, (**F**) IGF-2 receptor, (**G**) IRS-1, (**H**) IRS-2, and (**I**) IRS-4 by qRT-PCR analysis. Box plots depict group median (horizontal bar), 95% confidence interval limits (upper and lower box limits), and range (whiskers). Inter-group comparisons were made using Student *T*-tests. Significant *p*-values are indicated within the panels.

Sciatic nerve tissue was evaluated by qRT-PCR to examine expression of genes that regulate insulin and IGF signaling networks. All samples had detectable mRNA levels of insulin, IGF-1, and IGF-2 polypeptides, their corresponding receptors, and IRS-1, IRS-2, and IRS-4 (Figure 2). Among the polypeptide genes, IGF-2 was by far the most abundantly expressed, while insulin and IGF-1 were similarly low-level. In contrast, among the receptors, insulin receptor was most abundantly expressed, followed by IGF-2, then IGF-1 receptor. IRS-1 and IRS-2 were similarly abundant, while IRS-4 was expressed at much lower levels. Chronic ethanol feeding significantly reduced the mean mRNA levels of insulin, IGF-1, and IGF-2 polypeptides, IGF-1 receptor, and IRS-2 (Figure 2). In contrast, the mean mRNA levels of insulin and IGF-2 receptors, IRS-1, and IRS-4 were similar in the control and ethanol-exposed groups. 

### 3.5. Ethanol Effects on Insulin/IGF Signaling Molecules-ELISA Studies

To further characterize the effects of chronic ethanol exposure on insulin and IGF signaling networks, we measured insulin and IGF-1 receptors, IRS-1, IRS-2, and IRS-4 immunoreactivity in sciatic nerve tissue by direct binding ELISA. Results were normalized to protein content in the wells. Those studies demonstrated significantly reduced expression of the insulin and IGF-1 receptors, IRS-1, and IRS-4 in sciatic nerve tissue from ethanol-exposed relative to control rats (Table 4). It is noteworthy that the inter-group differences were prominent with respect to the receptors, but relatively modest (although statistically significant) with respect to IRS-1 and IRS-4 immunoreactivity. Therefore, the major negative impact of ethanol on insulin/IGF signaling in peripheral nerve appears to be mediated at the level of receptor expression.

**Table 4:** Effects of chronic ethanol feeding on insulin and IGF signaling mechanisms and protein expression in sciatic nerve. Long Evans rats were fed with isocaloric liquid diets containing 0% or 37% ethanol by caloric content. Immunoreactivity was measured by ELISA. Data reflect net specific binding in relative light units ± S.E.M. Inter-group comparisons (*N* = 8/group) were made by Student *T*-tests. INS-R = insulin receptor; IGF-1R = IGF-1 receptor; IRS = insulin receptor substrate; MAG = myelin-associated glycoprotein.

Protein	Control	Ethanol	*p*-Value
INS-R	37839.22 ± 2652.53	34612.68 ± 2319.60	2.41056 × 10^−5^
IGF-1R	39189.66 ± 2245.76	36973.84 ± 2254.17	0.000678
IRS-1	1254.47 ± 83.49	1220.34 ± 38.77	0.0379
IRS-2	1774.17 ± 201.19	1691.24 ± 148.64	0.0556
IRS-4	2022.31 ± 217.02	1798.44 ± 273.98	0.0015
MAG-1	21651.73 ± 1481.85	20425.26 ± 1157.55	0.001268
Tau	40046.09 ± 6460.38	35090.79 ± 5809.52	0.003782

### 3.6. Ethanol Effects on Peripheral Nerve Structural Proteins and Oxidative Stress

We next examined whether the ethanol-associated abnormalities in peripheral nerve function and insulin/IGF signaling mechanisms were associated with reductions in myelin associated glycoprotein-1 (MAG-1) and tau immunoreactivity, as indices of Schwann cell and neuronal integrity, respectively. Direct binding ELISAs using sciatic nerve tissue revealed significantly reduced mean levels of MAG-1 and tau in sciatic nerve tissue from ethanol-fed rats (Table 4). Therefore, like chronic alcohol-related brain and liver injury, experimental ALPN is associated with cellular degeneration and increased oxidative stress.

## 4. Discussion

This study was designed to characterize functional, morphologic, molecular, and biochemical abnormalities in an experimental animal model of ALPN. In previous studies, the Long Evans model of chronic ethanol feeding was demonstrated to have a robust and reproducible phenotype with respect to steatohepatitis and neurodegeneration, fetal alcohol spectrum disorders, and impaired placentation [26,29,44,47,50,51]. Likewise, the Long Evans rat model of chronic ethanol exposure has proven suitable for interrogating mechanisms of ALPN, and testing the efficacy of potential treatments for this disease. 

The major finding in this study was that in the absence of nutritional deficiencies, chronic heavy ethanol exposure can cause peripheral neuropathy characterized by predominantly slowing of nerve fiber conduction with subtle evidence of demyelination, together with some degree of axonal loss as demonstrated with histopathologic, morphometric, and molecular/biochemical analyses. In addition, this model of experimental ALPN was associated with impairments in the expression of genes and proteins that mediate intracellular signaling through insulin and IGF receptors and IRS molecules. Of note is that, these pathways play critical roles in mediating neuronal and Schwann cell survival, axonal and myelin maintenance, plasticity, and energy metabolism [30]. 

Since the liquid diets were isocaloric, replete with macro- and micronutrients including all essential vitamins, including thiamine, and the control and ethanol-exposed rats gained similar amounts of weight over the course of this study, it is likely that in this model, the ALPN was caused by neurotoxic or neurodegenerative effects of ethanol rather than nutritional deficiencies. The finding that ALPN can occur in the absence of nutritional deficiencies is consistent with observations in humans [8,9,25], and corroborates clinical trial data showing that thiamine repletion therapy does not significantly abate or reverse ALPN [9,52]. On the other hand, one cannot entirely exclude a role for organ/tissue thiamine deficiency in ALPN, particularly in humans, since alcohol impairs thiamine absorption through the gastrointestinal tract, thiamine utilization in tissues, and hepatic storage and phosphorylation of thiamine to thiamine pyrophosphate, the active form of the vitamin [15,16,17,18,19,25,53]. Therefore, although ALPN develops despite adequate nutrient intake, further research is needed to determine if impaired tissue utilization of thiamine is a co-factor in the pathogenesis of ALPN. In addition, studies are needed to determine the degree to which experimental thiamine deficiency exacerbates the phenotype of ALPN. The relevance of such studies is that, in order to optimally reverse or abate symptoms of ALPN, treatments for insulin/IGF resistance may have to be combined with active and continuous thiamine repletion therapy.

Our finding that the major effect of chronic ethanol exposure was to reduce conduction velocity due to peripheral nerve demyelination bears similarities and differences with respect to ALPN in humans. In humans, the electrophysiological and pathological features of ALPN mainly correspond to axonal degeneration that predominantly involves small fibers [5,8,9,25]. However, ALPN in humans can be associated with modest prolongation of distal latency, as well as pathological evidence of secondary demyelination [9,25], with evidence of large fiber degeneration as well [9,24]. In our rat model, despite electrophysiological evidence of prolonged conduction latency, the histopathological evidence of demyelination was modest. Conceivably, these effects of chronic ethanol exposure could have been mediated by functional impairment of neuromuscular junctions or nodes of Ranvier, or metabolic dysfunctions related to insulin/IGF resistance.

Another discordant result was that in our rat model, the only electrophysiological abnormalities detected were in motor and mixed (sciatic) nerves; in humans, ALPN is associated with burning pain, dysesthesias, and small fiber degeneration rather than motor dysfunction [5,8,9,24,25]. In humans, thiamine deficiency neuropathy is associated with motor impairment and degeneration of large axons [9,10]. The significantly smaller myofiber diameters measured in ethanol-fed rats was striking in relation to the minimal motor deficits observed by visual inspection. Although we did not weigh the muscles, there were no obvious differences in muscle bulk. One potential explanation for the small myofiber diameters and apparently similar muscle bulk is that the chronic injury led to sprouting and compensatory increase in myofiber density. Correspondingly, we detected myofiber splitting in histological sections of ethanol-exposed muscle. It is worth emphasizing that despite electrophysiological and histopathological evidence of peripheral neuropathy, the rats exhibited no detectable behavioral abnormalities. This suggests that substantial nerve degeneration can occur prior to the onset of symptoms. Therefore, highly sensitive tests may be needed to detect early signs of ALPN in humans because the onset of clinically significant symptoms may herald severe and irreversible peripheral nerve degeneration.

One potential explanation for these discrepancies is that in our rat model, ALPN may have been caused by combined effects of alcohol-mediated neurotoxicity and neurodegeneration, and possibly factors related to impaired thiamine transport and utilization. Alternatively, the outcomes of chronic ethanol exposures in rats may differ from those in humans due to the greater complexity of human lifestyles, bipedal versus quadripedal ambulation, and variability in the quality, time course, and episodic nature of alcohol consumed by humans versus experimental rats. A third possibility is that the methods used to detect impairments in nerve conduction were not sufficiently sensitive for evaluating functional impairments in small superficial nerve fibers. In support of the latter concept is the fact that axonal neuropathy, characterized by fiber loss, regenerative sprouting, and denervation myopathy, was detected in 1-micron thick sections, but not by electrophysiological measures. The failure to find functional abnormalities in sensory nerves, despite well-recognized clinical symptoms of ALPN in humans, together with discordant findings of axonal neuropathy by histopathologic but not nerve conduction studies, suggest that thorough characterization of ALPN with more sensitive and multi-pronged tools will likely be required for early diagnosis and treatment. 

Over the past several years, data have emerged illustrating that impaired insulin and IGF signaling mechanisms, as well as increased oxidative stress have critical roles in the pathogenesis of alcohol-related diseases of the brain and liver [27,28,29,45,54]. Once established, these pathophysiologic processes contribute to progressive cell loss, degeneration, and impaired organ/tissue function. In the brain, insulin/IGF signaling maintains neuronal and oligodendrocyte function, including the integrity of neuronal cytoskeleton and myelin homeostasis [30,45]. An established feature of alcohol-related brain disease, both in humans and experimental animal models, is brain insulin/IGF resistance accompanied by neuronal loss, white matter atrophy, and reduced myelin gene expression [27,28,36,37,45]. Similarly, Schwann cells, which produce peripheral nerve myelin, utilize IGF signaling for myelinogenesis and myelin maintenance [33,43,55]. However, little is known about the roles of insulin/IGF resistance and oxidative stress in Schwann cells as mediators of ALPN.

We performed qRT-PCR and ELISA studies on peripheral nerve tissues to determine if experimental ALPN was associated with impairments in insulin and IGF signaling mechanisms. The qRT-PCR analyses demonstrated that several of the genes and proteins that mediate insulin/IGF signaling were readily detected in peripheral nerve tissue, and that chronic ethanol feeding significantly reduced expression of insulin, IGF-I and IGF-2 polypeptides, IGF-1 receptor, and IRS-2 mRNA levels. ELISAs revealed significantly reduced expression of insulin and IGF-1 receptors, IRS-1, and IRS-4 proteins in peripheral nerve tissues from ethanol-fed rats. Although IRS-2 protein was also reduced in ethanol-fed rats, the difference from control only constituted a statistical trend. Altogether, these results suggest that ALPN is associated with both deficient expression insulin/IGF trophic factors, and impaired signaling through their corresponding receptors, and downstream through IRS proteins. These effects of alcohol are reminiscent of our findings in previous studies of alcohol-induced steatohepatitis, neurodegeneration, and placental insufficiency, and cerebellar hypoplasia associated with fetal alcohol spectrum disorders [26,28,45,47,50,51].

Consequences of impaired insulin/IGF signaling in the nervous system include neurodegeneration due to increased oxidative stress and inability to maintain neuronal and myelin structure and function. Correspondingly, we also demonstrated significantly reduced levels of MAG-1 and Tau expression in ethanol-exposed relative to control peripheral nerve tissue. Myelin and neuronal cytoskeletal proteins, including MAG-1 and Tau, are stimulated by insulin and/or IGF-1 [33,56,57]. The ethanol-associated reductions in local trophic factor and receptor expression, together with IRS molecules that transmit insulin/IGF signals, could account for the significant reductions in proteins needed to maintain myelin and axonal integrity. Therefore, ethanol-mediated impairments in insulin/IGF signaling mechanisms could account for both the demyelination and axonal degeneration in this experimental model of ALPN. Future studies will mechanistically determine the degree to which ALPN is specifically mediated by insulin/IGF resistance, and whether the disease can be ameliorated by treatment with insulin sensitizer agents such as peroxisome proliferator-activated receptor (PPAR) agonists.

## 5. Conclusions

This study demonstrates that experimental alcohol-related peripheral neuropathy can develop in the absence of nutritional deficiencies, including thiamine, and is characterized by (1) slowed conduction velocity due to predominantly demyelination, although a small component of axonal degeneration co-exists; (2) peripheral nerve insulin and IGF resistance, which occurs at multiple levels in the cascades, including at the insulin/IGF receptors, similar to the effects of ethanol on liver and brain; and (3) degeneration of myelin and axonal cytoskeletal proteins. We propose that alcohol-related peripheral neuropathy may be mediated by molecular and signal transduction abnormalities similar to those identified in alcoholic liver and brain degeneration.
